# What We Know and What We Don't Know About the Function of γδ T Cells

**DOI:** 10.1002/eji.70058

**Published:** 2025-09-15

**Authors:** Immo Prinz, Anja Meyer

**Affiliations:** ^1^ Institute of Systems Immunology University Medical Center Hamburg‐Eppendorf Hamburg Germany; ^2^ Institute of Immunology Hannover Medical School Hannover Germany

**Keywords:** T cells, γδ T cell function, γδ T cells, γδ TCR, γδ TCR ligands

## Abstract

γδ T cells, long regarded as unconventional relatives of αβ T cells, have emerged as pivotal players in immunity, with unique biology and therapeutic promise. Recent advances in single‐cell multiomics, refined mouse models, and human cohort studies have deepened insights into their TCR–ligand interactions, developmental pathways, and context‐dependent functions. This mini‐review synthesizes current understanding from structural studies of γδ TCR recognition and developmental regulation—including inborn errors of immunity—to adaptive‐like clonal expansions shaped by infection, aging, and environmental cues. It also highlights their dual roles in cancer, where subsets can exert potent cytotoxicity or promote tumor progression, and discusses strategies to optimize their antitumor potential through checkpoint blockade, metabolic modulation, and engineered receptors. Beyond immunity to malignancy, γδ T cells contribute to tissue homeostasis, repair, and regulation of inflammatory processes in diverse organs, influencing outcomes in neuroinflammation, autoimmunity, and fibrotic diseases. Together, these perspectives form the foundation of a special issue in the *European Journal of Immunology* (https://onlinelibrary.wiley.com/doi/toc/10.1002/(ISSN)1521‐4141.T‐cells) dedicated to advancing the understanding of γδ T cell biology and clinical potential.

## Graphical abstract

This mini‐review introduces a special issue on γδ T cells in the *European Journal of Immunology*. It summarizes how recent advances in single‐cell multiomics, mouse models, and human studies have clarified the developmental pathways, TCR–ligand interactions, and adaptive‐like responses of γδ T cells. A thorough understanding of the complex biology of these cells is necessary to unlock their full potential as targets for immunotherapy.

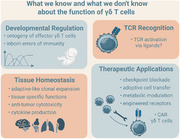

**Abbreviations**
BTN: butyrophilin
BTNL: butyrophilin‐like
CAR: chimeric antigen receptor
CDR3: complementarity‐determining region 3 (hyper‐diverse region, e.g., of a TCR)
IDO: indoleamine 2,3‐dioxygenase (tryptophan‐catabolizing enzyme)
PD‐1: programmed cell death protein 1
TIGIT: T‐cell immunoreceptor with immunoglobulin and ITIM domains
TRuC: TCR fusion construct

## 1 Introduction

**FIGURE 1:**
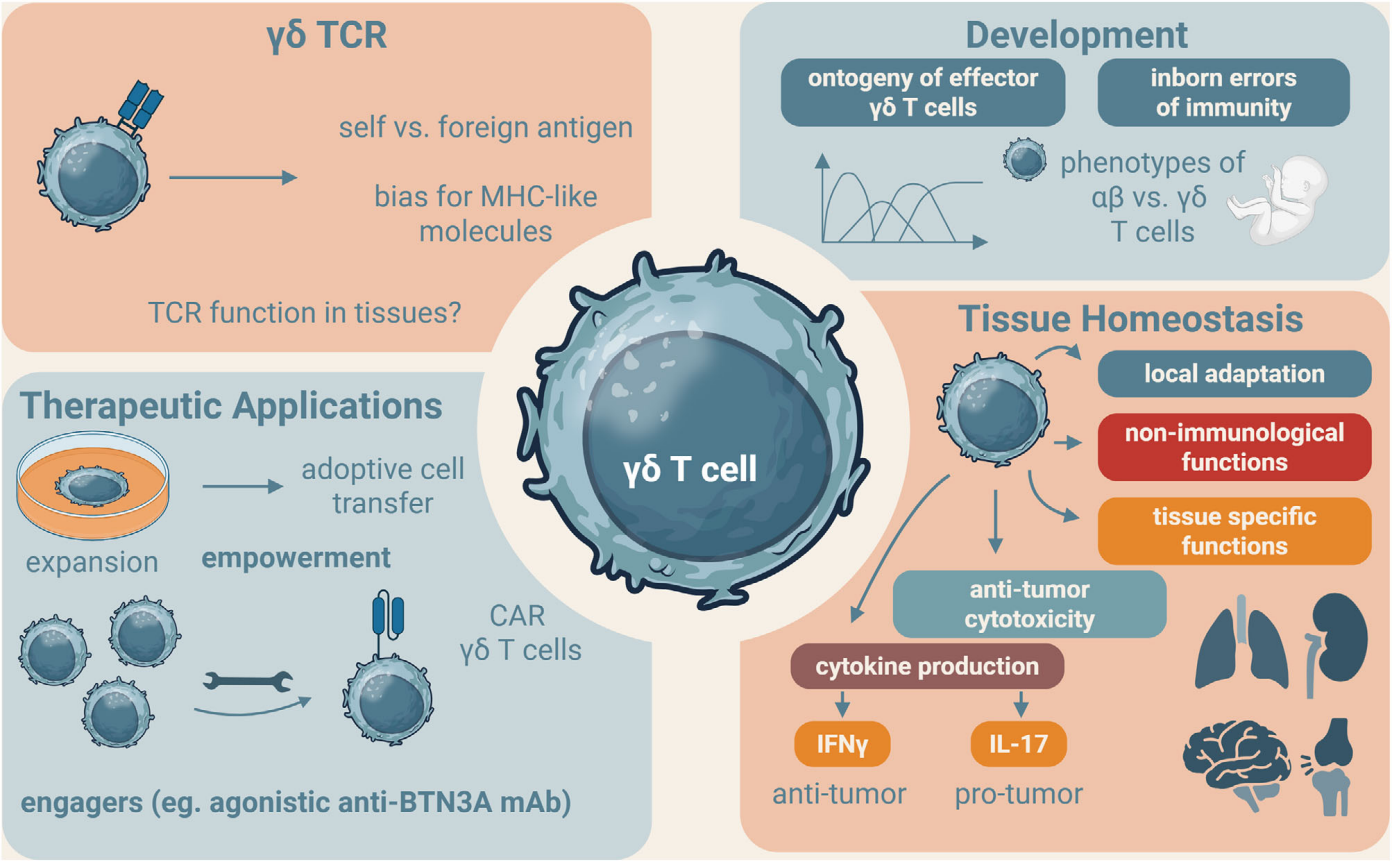
Current questions in γδ T cell research: understanding development, tissue function, and clinical application. This figure provides a schematic overview of the current questions in γδ T cell research. It covers cutting‐edge topics, including the unique features of γδ TCRs; the development of γδ T cells, including inborn errors of immunity; their specialized roles in tissue homeostasis, autoimmune diseases, and cancer immunity; and innovative strategies to harness γδ T cells for immunotherapy.

γδ T cells have long been considered an unusual companion to αβ T cells, with no apparent nonredundant functions in health and disease. However, the field has disproportionately profited from applying recent advances in single‐cell multiomics to refined mouse models and human cohort studies [1]. Therefore, it is a great timing and a pleasure to preface the special issue on γδ T cells in the *European Journal of Immunology* (https://onlinelibrary.wiley.com/doi/toc/10.1002/(ISSN)1521‐4141.T‐cells), which comprises twelve reviews and three original research works that feature many aspects of what we know and don't know about γδ T cell function. These contributions cover the most exciting topics, ranging from the distinctive TCR characteristics of the γδ TCR and γδ T cell development, including inborn errors of immunity, to their specific functions in tissue homeostasis, autoimmunity, and anticancer immunity, as well as exploring new ways to empower γδ T cells for immunotherapy (Figure 1).

## 2 Functions and Ligands of γδ T Cell Receptors

The TCRs of γδ T cells, namely γδ TCRs, are composed of gamma and delta chains instead of alpha and beta chains in αβ T cells. Currently, “What is recognized by the γδ TCR?” may still be the most challenging question in the field (reviewed in detail previously in [2, 3]). It is assumed that the majority of γδ TCRs can recognize diverse epitopes of their ligands in a manner reminiscent of how antibodies recognize surface antigens [4, 5]. In addition, certain invariant and thus rather “innate” γδ TCRs use germline‐encoded TCR Vγ regions to interact with specific BTN/BTNL family members in a superantigen‐like fashion. The most prominent example is the recognition of phosphoantigen‐binding BTN3A1 and BT2A1 molecules by T cells with invariant Vγ9Vδ2^+^ TCRs [6, 7, 8], for which the molecular basis is revisited by Herrmann and Karunakaran in this issue [9]. Importantly, γδ TCRs are neither restricted to recognizing peptide/MHC complexes nor are they strictly selected against recognizing self‐molecules [10]. At the 11th International γδ T Cell Conference in Toronto in May 2025, however, Erin Adams suggested that γδ T cells should not generally be described as “not MHC‐restricted” because up to 20% of γδ TCRs may react with MHC or MHC‐related molecules in some way. It was agreed that future trials will have to determine whether this estimation is in the right ballpark. Indeed, of few interactions between γδ TCRs and cognate ligands that have been verified biochemically or structurally, most show a bias for binding MHC‐like surface proteins such as T22 and CD1d [11, 12]. However, recognition of MHC Class I or Class II molecules by γδ TCRs can be dependent [13] or independent [14] of presented peptides, and interactions are often not involving the CDR3 regions of the γδ TCR [15].

To investigate how certain γδ TCRs instruct the phenotype of maturing tissue‐specific and functional subsets of γδ T cells in thymus and periphery, Winkler and colleagues have recently generated the first in situ transgenic TCR knock‐in mouse with a fixed monoclonal TCR delta chain [16]. Here, they review their surprising observation that a single Vδ1 chain can support the development of basically all known mouse γδ T cell subsets and further discuss novel implications regarding the environmental cues and the transcriptional regulation that drive γδ T cells toward a CD8αα^+^ intraepithelial lymphocyte phenotype [17]. As the γδ TCR defines the γδ T cell lineage, it is obviously required for γδ T cell development [18]. However, the development and function of αβ and γδ T cells seem to be differentially affected by inborn errors of immunity. In their review, Sagar and Ehl elucidate the impact of inborn genetic errors as well as induced variations on the development of human γδ T cells [19]. Along this line, earlier work from the Malissen lab showed that proper γδ TCR signaling is required for a “quality control” positive selection of γδ T cells [20, 21]. In contrast, it is less clear how important the γδ TCR is for γδ T cell function in the periphery [22]. Some intraepithelial γδ T cells are constantly receiving signals through their γδ TCR [23, 24]. Yet, information about γδ T cells losing these signals via genetic ablation is sparse [25, 26].

Advocating for an instructive role of the γδ TCR for the differentiation of mature γδ T cells in the periphery, high‐throughput γδ TCR analyses revealed that most humans acquire highly expanded persisting γδ clones at some point in their life [27, 28]. These findings are consistent with an adaptive‐like immune response in all γδ T cells that do not use invariant Vγ9Vδ2^+^ TCRs, which are associated with a more innate immune response. This has stimulated research into the conditions under which γδ T cells expand. Focusing on human γδ T cell population dynamics from birth to old age, Ravens and Tolosa discuss that recent multiomic approaches have advanced our understanding of γδ T‐cell development in the thymus and their functional adaptation to the microbial environment in the periphery [29]. In addition, original research from the Vermijlen group is contributing new, exciting data to the general understanding of the life cycle of human γδ T cells. This research reports how the human γδ TCR repertoire is shaped by sepsis in an age‐ and pathogen‐dependent manner [30], and how maternal administration of probiotics augments IL‐17‐committed γδ T cells in newborns [31].

## 3 γδ T Cells Against Cancer

The intrinsic ability of γδ T cells to recognize and attack malignancies independently of tumor antigen presentation by MHC molecules exposes their translational potential for cancer treatment in situations where neoantigen‐specific αβ T cells are inefficient. This has stimulated growing translational interest in γδ T cells, bringing them out of the shadows of αβ T cells and into the spotlight [32]. Although promising results have emerged from clinical trials investigating the use of γδ T cells in anticancer therapy, some problems remain unresolved and further research is necessary [33].

In particular, current research investigating how antitumor reactivity of γδ T cells is achieved focuses on the role of the γδ TCR. Seminal studies reported that individual Vδ1^+^ γδ TCRs seem to be specifically reactive and potentially protective against hitherto unknown ligands expressed by limited sets of cancer types [34]. However, blocking the checkpoints PD‐1 and TIGIT may be required to unlock the full antitumor cytotoxicity of Vδ1^+^ γδ T cells infiltrating melanoma, Merkel cell carcinoma, or colorectal cancer [35, 36]. Indeed, adoptive γδ T cell therapy seems to work best in combination with immune checkpoint blockade and modulation of cytotoxic receptors [37, 38].

The translation of γδ T cells to therapies against tumors is further complicated by observations that some γδ T cell subsets may even promote tumor growth. Fiala and colleagues address these issues by discussing the pro‐ and antitumorigenic functions of γδ T cells [39]. They summarize the mechanisms by which γδ T cells exert seemingly contradictory effector functions due to their preferential production of the antitumor IFN‐γ or pro‐tumor IL‐17A, and cast light on the functions of human γδ T cells in cancer. Furthermore, it is important to explore and eventually modify the mechanisms by which γδ T cells infiltrate solid tumors and how the tumor microenvironment influences γδ T cell function, e.g., through IDO overexpression and galectin crosstalk. Here, Wistuba‐Hamprecht and colleagues review the spatial dimension for the proper function and organization of tumor‐invasive human γδ T cells [40]. γδ T cells may be specifically manipulated and engineered to perform better in antitumor assays and ultimately in antitumor therapies. Concepts to enhance the antitumor effector functions and ensure optimized clinical efficacy include culturing γδ T cells in the presence of vitamin C, as explained by Kabelitz and colleagues [41]. Other concepts propose to equip in vitro expanded γδ T cells with classical chimeric antigen receptors (CARs), or to use tumor‐reactive γδ TCRs as “natural CARs.” Accordingly, extending the horizon of how γδ T cells still represents an unearthed treasure for immunotherapy. To this end, Schamel and colleagues review the potential of CAR γδ T cells and TRuC γδ T cells. They also provide a detailed discussion of the current implications, safety, and obstacles of using engineered γδ T cells in cancer therapy [42].

## 4 Tissue‐Specific Functions of γδ T Cells

In addition to their anticancer reactivity, established functions of γδ T cells include warranting tissue homeostasis, surveillance, and regeneration. They also have non‐immunological functions, such as thermogenesis and modulation of cognitive processes via γδ T cells residing in the central nervous system [43]. In this special issue, Bulgur and colleagues review γδ T cell functions in the central nervous system, highlighting upcoming challenges in the field and providing new insights into potential therapeutic strategies. The authors speculate about the molecular mechanisms behind their findings on the contribution of γδ T cells to neuropathophysiology. They recently pioneered the identification of an embryonically derived Vγ6^+^ γδ T cell subset that infiltrates the meninges from birth and comprises most IL‐17‐producing cells [44]. The authors further suggest that γδ T cells may be responsible for triggering or preventing inflammatory responses in neurodegenerative diseases as well as neuropsychiatric disorders [45].

Next are reviews on the roles of γδ T cells in autoinflammatory spondylarthritides [46], immune‐mediated kidney diseases (including glomerulonephritis) [47], recovery of the ischemic brain after stroke [48], and a study on the role of γδ T cells in the pathobiology of experimental lung fibrogenesis [49]. These reviews complement the section on the functions of γδ T cells in tissue homeostasis and regeneration. γδ T cells have also been implicated in chronic inflammatory skin diseases such as psoriasis. In this disease, IL‐17‐producing dermal γδ T cells switch toward aerobic glycolysis, contributing to disease severity and relapse [50]. In models of inflammatory bowel disease, IL‐17A‐producing γδ T cells promote intestinal inflammation in an IL‐23‐dependent manner. However, under steady‐state conditions, these cells can also exhibit protective functions in epithelial repair [51]. These contributions highlight the central role of γδ T cells in Type 3 immunity and inflammatory diseases along the IL‐23/IL‐17 cytokine axis. While γδ T cells are a major, if not the primary, source of IL‐17A and IL‐17F in mouse tissues, IL‐17 cytokine production by γδ T cells appears to be more tightly regulated in humans and restricted to specific subsets such as Vγ9Vδ2 T cells under inflammatory conditions [52].

In conclusion, this special issue offers substantial, novel insights into mouse and human γδ T cell development and the role of γδ T cells in various inflammatory diseases. At the same time, it highlights their potential for immunotherapy against cancer.

## Conflicts of Interest

The authors declare no conflicts of interest.

## Peer Review

The peer review history for this article is available at https://publons.com/publon/10.1002/eji.70058.

## Data Availability

Data sharing is not applicable to this article as no datasets were generated or analyzed during the current study.

## References

[1] D. Inacio , T. Amado , A. Pamplona , et al., “Signature Cytokine‐Associated Transcriptome Analysis of Effector γδ T Cells Identifies Subset‐Specific Regulators of Peripheral Activation,” Nature Immunology 26 (2025): 497–510. 39881001 10.1038/s41590-024-02073-8 PMC11876068

[2] B. E. Willcox and C. R. Willcox , “γδ TCR Ligands: The Quest to Solve a 500‐Million‐Year‐Old Mystery,” Nature Immunology 20 (2019): 121–128. 30664765 10.1038/s41590-018-0304-y

[3] M. Deseke and I. Prinz , “Ligand Recognition by the γδ TCR and Discrimination Between Homeostasis and Stress Conditions,” Cellular & Molecular Immunology 17 (2020): 914–924. 32709926 10.1038/s41423-020-0503-y PMC7608190

[4] C. L. Sok , J. Rossjohn , and B. S. Gully , “The Evolving Portrait of γδ TCR Recognition Determinants,” Journal of Immunology 213 (2024): 543–552. 10.4049/jimmunol.2400114 PMC11335310 39159405

[5] C. R. Willcox , F. Mohammed , and B. E. Willcox , “The Distinct MHC‐Unrestricted Immunobiology of Innate‐Like and Adaptive‐Like Human γδ T Cell Subsets‐Nature's CAR‐T Cells,” Immunological Reviews 298 (2020): 25–46. 33084045 10.1111/imr.12928

[6] T. S. Fulford , C. Soliman , R. G. Castle , et al., “Vγ9Vδ2 T Cells Recognize Butyrophilin 2A1 and 3A1 Heteromers,” Nature Immunology 25 (2024): 1355–1366. 39014161 10.1038/s41590-024-01892-z

[7] M. M. Karunakaran , C. R. Willcox , M. Salim , et al., “Butyrophilin‐2A1 Directly Binds Germline‐Encoded Regions of the Vγ9Vδ2 TCR and Is Essential for Phosphoantigen Sensing,” Immunity 52 (2020): 487–498.e6. 32155411 10.1016/j.immuni.2020.02.014 PMC7083227

[8] M. Rigau , S. Ostrouska , T. S. Fulford , et al., “Butyrophilin 2A1 Is Essential for Phosphoantigen Reactivity by γδ T Cells,” Science 367 (2020): eaay5516. 31919129 10.1126/science.aay5516

[9] T. Herrmann and M. M. Karunakaran , “Phosphoantigen Recognition by Vγ9Vδ2 T Cells,” European Journal of Immunology 54 (2024): e2451068. 39148158 10.1002/eji.202451068

[10] H. Schild , N. Mavaddat , C. Litzenberger , et al., “The Nature of Major Histocompatibility Complex Recognition by Gamma Delta T Cells,” Cell 76 (1994): 29–37. 8287478 10.1016/0092-8674(94)90170-8

[11] E. J. Adams , Y. H. Chien , and K. C. Garcia , “Structure of a γδ T Cell Receptor in Complex With the Nonclassical MHC T22,” Science 308 (2005): 227–231. 15821084 10.1126/science.1106885

[12] A. M. Luoma , C. D. Castro , T. Mayassi , et al., “Crystal Structure of Vδ1 T Cell Receptor in Complex With CD1d‐Sulfatide Shows MHC‐Like Recognition of a Self‐Lipid by Human γδ T Cells,” Immunity 39 (2013): 1032–1042. 24239091 10.1016/j.immuni.2013.11.001 PMC3875342

[13] P. M. Benveniste , S. Roy , M. Nakatsugawa , et al., “Generation and Molecular Recognition of Melanoma‐Associated Antigen‐Specific Human γδ T Cells,” Science Immunology 3 (2018): eaav4036. 30552102 10.1126/sciimmunol.aav4036

[14] M. Deseke , F. Rampoldi , I. Sandrock , et al., “A CMV‐Induced Adaptive Human Vδ1 + γδ T Cell Clone Recognizes HLA‐DR,” Journal of Experimental Medicine 219 (2022): e20212525. 35852466 10.1084/jem.20212525 PMC9301659

[15] J. Le Nours , N. A. Gherardin , S. H. Ramarathinam , et al., “A Class of γδ T Cell Receptors Recognize the Underside of the Antigen‐Presenting Molecule MR1,” Science 366 (2019): 1522–1527. 31857486 10.1126/science.aav3900

[16] A. M. Hahn , L. Vogg , S. Brey , et al., “A Monoclonal Trd Chain Supports the Development of the Complete Set of Functional γδ T Cell Lineages,” Cell Reports 42 (2023): 112253. 36920908 10.1016/j.celrep.2023.112253

[17] L. Vogg and T. H. Winkler , “Nurturing the Phenotype: Environmental Signals and Transcriptional Regulation of Intestinal γδ T Cells,” European Journal of Immunology (2024): e2451076. 39136644 10.1002/eji.202451076

[18] I. Prinz , B. Silva‐Santos , and D. J. Pennington , “Functional Development of γδ T Cells,” European Journal of Immunology 43 (2013): 1988–1994. 23928962 10.1002/eji.201343759

[19] Sagar and S. Ehl , “γδ T Cells and Inborn Errors of Immunity,” European Journal of Immunology 55 (2025): e51457. 40556329 10.1002/eji.202451457 PMC12188109

[20] I. Prinz , A. Sansoni , A. Kissenpfennig , L. Ardouin , M. Malissen , and B. Malissen , “Visualization of the Earliest Steps of γδ T Cell Development in the Adult Thymus,” Nature Immunology 7 (2006): 995–1003. 16878135 10.1038/ni1371

[21] S. Nunez‐Cruz , E. Aguado , S. Richelme , et al., “LAT Regulates γδ T Cell Homeostasis and Differentiation,” Nature Immunology 4 (2003): 999–1008. 12970761 10.1038/ni977

[22] M. Wencker , G. Turchinovich , R. Di Marco Barros , et al., “Innate‐Like T Cells Straddle Innate and Adaptive Immunity by Altering Antigen‐Receptor Responsiveness,” Nature Immunology 15 (2014): 80–87. 24241693 10.1038/ni.2773 PMC6485477

[23] F. H. Malinarich , E. Grabski , T. Worbs , et al., “Constant TCR Triggering Suggests That the TCR Expressed on Intestinal Intraepithelial γδ T Cells Is Functional In Vivo,” European Journal of Immunology 40 (2010): 3378–3388. 21108461 10.1002/eji.201040727

[24] G. Chodaczek , V. Papanna , M. A. Zal , and T. Zal , “Body‐Barrier Surveillance by Epidermal γδ TCRs,” Nature Immunology 13 (2012): 272–282. 22327568 10.1038/ni.2240 PMC3288780

[25] D. R. McKenzie , R. Hart , N. Bah , et al., “Normality Sensing Licenses Local T Cells for Innate‐Like Tissue Surveillance,” Nature Immunology 23 (2022): 411–422. 35165446 10.1038/s41590-021-01124-8 PMC8901436

[26] B. Zhang , J. Wu , Y. Jiao , et al., “Differential Requirements of TCR Signaling in Homeostatic Maintenance and Function of Dendritic Epidermal T Cells,” Journal of Immunology 195 (2015): 4282–4291. 10.4049/jimmunol.1501220 PMC4610875 26408667

[27] S. Ravens , C. Schultze‐Florey , S. Raha , et al., “Human γδ T Cells Are Quickly Reconstituted After Stem‐Cell Transplantation and Show Adaptive Clonal Expansion in Response to Viral Infection,” Nature Immunology 18 (2017): 393–401. 28218745 10.1038/ni.3686

[28] M. S. Davey , C. R. Willcox , S. P. Joyce , et al., “Clonal Selection in the Human Vδ1 T Cell Repertoire Indicates γδ TCR‐Dependent Adaptive Immune Surveillance,” Nature Communications 8 (2017): 14760. 10.1038/ncomms14760 PMC5337994 28248310

[29] S. Ravens and E. Tolosa , “Expansion of Human γδ T Cells in Periphery: Lessons Learned From Development, Infections, and Compromised Thymic Function,” European Journal of Immunology 54 (2024): e2451073. 39194409 10.1002/eji.202451073

[30] E. Giannoni , G. Sanchez Sanchez , I. Verdebout , et al., “Sepsis Shapes the Human γδ TCR Repertoire in an Age‐ and Pathogen‐Dependent Manner,” European Journal of Immunology 54 (2024): e2451190. 39072722 10.1002/eji.202451190

[31] Y. Tafesse , A. Kohler , G. Sanchez Sanchez , et al., “Maternal Administration of Probiotics Augments IL17‐Committed γδ T Cells in the Newborn Lung,” European Journal of Immunology 55 (2025): e202451051. 40259457 10.1002/eji.202451051

[32] Z. Sebestyen , I. Prinz , J. Dechanet‐Merville , B. Silva‐Santos , and J. Kuball , “Translating Gammadelta (γδ) T Cells and Their Receptors Into Cancer Cell Therapies,” Nature Reviews Drug Discovery 19 (2020): 169–184. 31492944 10.1038/s41573-019-0038-z

[33] A. Hayday , J. Dechanet‐Merville , J. Rossjohn , and B. Silva‐Santos , “Cancer Immunotherapy by γδ T Cells,” Science 386 (2024): eabq7248. 39361750 10.1126/science.abq7248 PMC7616870

[34] M. J. Maeurer , D. Martin , W. Walter , et al., “Human Intestinal Vδ1 + Lymphocytes Recognize Tumor Cells of Epithelial Origin,” Journal of Experimental Medicine 183 (1996): 1681–1696. 8666926 10.1084/jem.183.4.1681 PMC2192504

[35] S. C. Lien , D. Ly , S. Y. C. Yang , et al., “Tumor Reactive γδ T Cells Contribute to a Complete Response to PD‐1 Blockade in a Merkel Cell Carcinoma Patient,” Nature Communications 15 (2024): 1094. 10.1038/s41467-024-45449-y PMC10848161 38321065

[36] V. Stary , R. V. Pandey , J. List , et al., “Dysfunctional Tumor‐Infiltrating Vδ1 + T Lymphocytes in Microsatellite‐Stable Colorectal Cancer,” Nature Communications 15 (2024): 6949. 10.1038/s41467-024-51025-1 PMC11322529 39138181

[37] R. Blanco‐Dominguez , L. Barros , M. Carreira , et al., “Dual Modulation of Cytotoxic and Checkpoint Receptors Tunes the Efficacy of Adoptive Delta One T Cell Therapy Against Colorectal Cancer,” Nature Cancer 6 (2025): 1056–1072. 40240620 10.1038/s43018-025-00948-9 PMC12202480

[38] S. Mensurado , C. Condeco , D. Sanchez‐Martinez , et al., “CD155/PVR Determines Acute Myeloid Leukemia Targeting by Delta One T Cells,” Blood 143 (2024): 1488–1495. 38437507 10.1182/blood.2023022992 PMC11033583

[39] G. J. Fiala , J. Lucke , and S. Huber , “Pro‐ and Antitumorigenic Functions of γδ T Cells,” European Journal of Immunology 54 (2024): e2451070. 38803018 10.1002/eji.202451070

[40] K. Wistuba‐Hamprecht , H. H. Oberg , and D. Wesch , “Function and Spatial Organization of Tumor‐Invasive Human γδ T Cells‐What Do We Know?,” European Journal of Immunology 55 (2025): e202451075. 39623788 10.1002/eji.202451075 PMC11739682

[41] D. Kabelitz , L. Cierna , C. Juraske , M. Zarobkiewicz , W. W. Schamel , and C. Peters , “Empowering γδ T‐cell Functionality With Vitamin C,” European Journal of Immunology 54 (2024): e2451028. 38616772 10.1002/eji.202451028

[42] W. W. Schamel , M. Zinchenko , T. Nguyen , B. Fehse , P. S. Briquez , and S. Minguet , “The Potential of γδ CAR and TRuC T Cells: An Unearthed Treasure,” European Journal of Immunology 54 (2024): e2451074. 39192467 10.1002/eji.202451074

[43] J. C. Ribot , N. Lopes , and B. Silva‐Santos , “γδ T Cells in Tissue Physiology and Surveillance,” Nature Reviews Immunology 21 (2021): 221–232. 10.1038/s41577-020-00452-4 33057185

[44] M. Ribeiro , H. C. Brigas , M. Temido‐Ferreira , et al., “Meningeal γδ T Cell‐Derived IL‐17 Controls Synaptic Plasticity and Short‐Term Memory,” Science Immunology 4 (2019): eaay5199. 31604844 10.1126/sciimmunol.aay5199 PMC6894940

[45] D. Bulgur , R. M. Moura , and J. C. Ribot , “Key Actors in Neuropathophysiology: The Role of γδ T Cells,” European Journal of Immunology 54 (2024): e2451055. 39240039 10.1002/eji.202451055 PMC11628923

[46] A. Meyer , “Illuminating the Impact of γδ T Cells in Man and Mice in Spondylarthritides,” European Journal of Immunology 54 (2024): e2451071. 39077953 10.1002/eji.202451071

[47] A. Waterholter , C. F. Krebs , and U. Panzer , “γδ T Cells in Immune‐Mediated Kidney Disease,” European Journal of Immunology 54 (2024): e2451069. 39289824 10.1002/eji.202451069 PMC11628881

[48] M. Piepke , A. Jander , N. Gagliani , and M. Gelderblom , “IL‐17A‐Producing γδ T Cells: A Novel Target in Stroke Immunotherapy,” European Journal of Immunology 54 (2024): e2451067. 39396374 10.1002/eji.202451067 PMC11628885

[49] M. T. Moog , M. Baltes , T. Ropke , et al., “Innate T‐Cell‐Derived IL‐17A/F Protects From Bleomycin‐Induced Acute Lung Injury but Not Bleomycin or Adenoviral TGF‐beta1‐Induced Lung Fibrosis in Mice,” European Journal of Immunology 54 (2024): e2451323. 39235361 10.1002/eji.202451323 PMC11628887

[50] Y. S. Kao , M. Lauterbach , A. Lopez Krol , et al., “Metabolic Reprogramming of Interleukin‐17‐Producing γδ T Cells Promotes ACC1‐Mediated De Novo Lipogenesis Under Psoriatic Conditions,” Nature Metabolism 7 (2025): 966–984. 10.1038/s42255-025-01276-z PMC12116387 40360755

[51] J. S. Lee , C. M. Tato , B. Joyce‐Shaikh , et al., “Interleukin‐23‐Independent IL‐17 Production Regulates Intestinal Epithelial Permeability,” Immunity 43 (2015): 727–738. 26431948 10.1016/j.immuni.2015.09.003 PMC6044435

[52] P. H. Papotto , A. Reinhardt , I. Prinz , and B. Silva‐Santos , “Innately Versatile: γδ17 T Cells in Inflammatory and Autoimmune Diseases,” Journal of Autoimmunity 87 (2018): 26–37. 29203226 10.1016/j.jaut.2017.11.006

